# A clustered controlled trial of the implementation and effectiveness of a medical home to improve health care of people with serious mental illness: study protocol

**DOI:** 10.1186/s12913-018-3237-0

**Published:** 2018-06-07

**Authors:** Alexander S. Young, Amy N. Cohen, Evelyn T. Chang, Anthony W. P. Flynn, Alison B. Hamilton, Rebecca Oberman, Merlyn Vinzon

**Affiliations:** 1 VA Greater Los Angeles Healthcare System, MIRECC, 11301 Wilshire Blvd., 210A, Los Angeles, CA 90073 USA; 2 Department of Psychiatry, Geffen School of Medicine, UCLA, 10920 Wilshire Blvd., Suite 300, Los Angeles, CA 90024 USA; 3 Department of Medicine, Geffen School of Medicine, UCLA, Los Angeles, CA 90095 USA

**Keywords:** Severe mental illness, Primary care, Patient aligned care team, Person-centered medical home, Mental health care, Cardiovascular disease, Quality improvement, Comparative effectiveness, Patient-centered care, Care management

## Abstract

**Background:**

People with serious mental illness (SMI) die many years prematurely, with rates of premature mortality two to three times greater than the general population. Most premature deaths are due to “natural causes,” especially cardiovascular disease and cancer. Often, people with SMI are not well engaged in primary care treatment and do not receive high-value preventative and medical services. There have been numerous efforts to improve this care, and few controlled trials, with inconsistent results. While people with SMI often do poorly with usual primary care arrangements, research suggests that integrated care and medical care management may improve treatment and outcomes, and reduce treatment costs.

**Methods:**

This hybrid implementation-effectiveness study is a prospective, cluster controlled trial of a medical home, the SMI Patient-Aligned Care Team (SMI PACT), to improve the healthcare of patients with SMI enrolled with the Veterans Health Administration. The SMI PACT team includes proactive medical nurse care management, and integrated mental health treatment through regular psychiatry consultation and a collaborative care model. Patients are recruited to receive primary care through SMI PACT based on having a serious mental illness that is manageable with treatment, and elevated risk for hospitalization or death. In a site-level prospective controlled trial, this project studies the effect, relative to usual care, of SMI PACT on provision of appropriate preventive and medical treatments, health-related quality of life, satisfaction with care, and medical and mental health treatment utilization and costs. Research includes mixed-methods formative evaluation of usual care and SMI PACT implementation to strengthen the intervention and assess barriers and facilitators. Investigators examine relationships among organizational context, intervention factors, and patient and clinician outcomes, and identify patient factors related to successful patient outcomes.

**Discussion:**

This will be one of the first controlled trials of the implementation and effectiveness of a patient centered medical home for people with serious mental illness. It will provide information regarding the value of this strategy, and processes and tools for implementing this model in community healthcare settings.

**Trial registration:**

ClinicalTrials.gov, NCT01668355. Registered August 20, 2012.

## Background

People with serious mental illness (SMI) experience much worse physical health outcomes than the rest of the population [1, 2]. People with SMI die many years prematurely, with rates of premature mortality two to three times greater than the general population. Most premature deaths are due to “natural causes,” especially cardiovascular respiratory disease and cancer. This disparity results from challenges at multiple levels. At the patient level, cognitive symptoms, decreased motivation, and social disadvantage impair patients’ ability to self-manage medical illnesses and navigate healthcare systems; and smoking and obesity are more common. At the provider level, mental health clinicians can have limited competency providing medical and preventive services and treatments, primary care clinicians can have limited competency with psychiatric illness and substance use disorders, and stigma is common. At the organizational level, systems often lack medical care management, or shared treatment and effective partnerships between primary care (PC) and mental health [3–5]. Often, people with SMI are not well engaged in primary care and do not receive high-value preventative and medical services. Given these intersecting challenges, advances are needed to improve healthcare for people with SMI. There have been numerous efforts to improve this care, and few controlled trials, with inconsistent results [6–8]. While people with SMI often do poorly with usual primary care arrangements, research suggests that integrated care and medical care management could improve treatment and outcomes, and reduce treatment costs [9–12].

The Veterans Health Administration (VA) has undertaken a major initiative to improve primary care for Veterans with mental illness via Primary Care-Mental Health Integration (PC-MHI). Since 2008, the PC-MHI initiative has sought to improve Veterans’ mental health conditions by co-locating mental health clinicians in primary care, and making care management services available in primary care for common psychiatric disorders. PC-MHI has focused on patients with depression and anxiety, with a goal of managing these patients within primary care and reserving specialty mental health care for patients with more advanced psychiatric needs. In other efforts, primary care clinicians have, at some VA medical centers, been co-located within specialty mental health settings to better support Veterans with SMI. However, this co-location has not been implemented widely, and researchers have found inconsistent effects on care process and outcomes [13, 14]. Patients with SMI are among the most complex patients medically, and co-located resources have not always responded to this complexity. Co-location of services may be necessary, but insufficient, as treatment processes require tailoring to the needs of people with SMI and collaboration between mental health and primary care clinicians to formulate treatment plans. Medical care management models can address these needs by improving access to preventive services and treatments and potentially reduce costs in populations with SMI [4].

VA primary care has been reorganized via dissemination of a Patient Aligned Care Team (PACT) model derived from the Patient Centered Medical Home (PCMH) concept [15, 16]. When fully implemented, the PACT model includes team-based care, care coordination, an emphasis on access, and the use of clinical registry data to proactively manage populations. The SMI PACT model builds on the PCMH model to tailor and intensify medical care management and integration of psychiatric care for patients with SMI. Further, SMI-PACT aims to maximize the delivery of preventive and evidence-based medical services [17]. SMI-PACT protocols focus on primary care team structure, point of care, patient outreach, panel management, and integration of psychiatric care into the team.

The usual VA PACT model consists of one full-time primary care provider (physician, nurse practitioner or physician assistant), supported by Registered Nurses (RNs), Licensed Practical Nurses (LPNs), pharmacists, medical assistants, health technicians, and medical clerks [15, 18]. Expected panel sizes for VA primary care providers are in the range of 1000 to 1400 patients. In SMI PACT, the clinical model consists of a teamlet with one 25% primary care physician, one half-time nurse care manager (RN), a consulting psychiatrist, and a clerical associate pool shared with other teamlets. Consistent with VA directives for high need specialty populations, SMI-PACT patient panels are smaller (*n* = 300–700 per full-time primary care provider) to accommodate an increase in the standard visit length from 20 to 30 min. Extra time is needed to allow for multiple, complex medical comorbidities. Also, time is required for proactive phone panel management, “desktop medicine” to respond to calls and secure patient messages, and time to contact other clinics and labs. All staff functions to their highest capability.

At the point of care, the registered nurse is the medical care manager for the patient panel as well as the communications hub of the team. This clinician maintains close communication with clerical associates and facilitates coordination within the team. To the highest degree possible, patients are active collaborators in their treatment plan, which is jointly owned by the PACT teamlet and patient. In addition to providing routine primary care services, the primary care physician works with the psychiatrist to resolve any conflict between patients’ illnesses and treatments (e.g., weight gain from antipsychotic medications). Patients are encouraged to use secure email to contact the team, complementing (or in lieu of) in-person visits with goals of 1/3 phone, 1/3 secure message, 1/3 in-person visits.

Patient outreach is systematized. Scripted messages are delivered to each patient to describe the new care model, clinicians on their team, their role in supporting this change, and potential care improvements. Patients who are newly assigned to the team receive a welcoming call within one month of assignment to encourage a strong clinician-patient working relationship [19]. Teamlet members contact patients to address issues. Lab tests are systematically reviewed and results reported to patients in a timely manner. Time is dedicated weekly meeting with psychiatric staff. When possible, same-day and walk-in appointments are made available to the nurse and the provider.

To improve care coordination and make most efficient use of clinician resources, whenever possible all patient care, including mental health, is provided within the SMI PACT team. Using a collaborative care approach, SMI PACT provides psychiatric care when the patient’s usual psychiatrist considers the patient to be psychiatrically stable with low risk, the patient does not require specialty psychiatric services, and this is consistent with patient preferences. Procedures are established between the SMI-PACT teamlet and specialty mental health teams regarding patient assignment and communication.

### Primary aims

The first aim is to implement and study the adapted SMI-PACT model in a site-level controlled trial with the goal of improving care conditions and outcomes for Veterans with SMI. The primary care medical home model is adapted to meet the needs of patients with SMI, and project leadership partners with one VA healthcare center to implement SMI-PACT. The second aim is to study the effect, relative to usual care, of SMI-PACT on the provision of appropriate preventive and medical treatments, and patient health-related quality of life and satisfaction with care. The effect on medical and mental health treatment utilization is studied, and costs are examined in exploratory analyses. The third aim is to use mixed methods to conduct a formative evaluation of usual care and SMI-PACT implementation to strengthen the intervention; to assess acceptability of the SMI-PACT model, and barriers and facilitators to its implementation; to investigate relationships among organizational context, intervention factors, and patient and clinician outcomes; and to identify patient factors related to successful patient outcomes.

## Methods and Design

### Study design

This study is a site-level, clustered controlled trial at three VA medical centers. All facilities have adopted the PC-MHI and PACT models. The SMI PACT model is implemented at one site. The other two sites remain with usual care. A random sample of 340 patients (170 intervention, 170 control) are recruited. Potentially eligible patients are identified using routine VA administrative data. Inclusion criteria include having serious mental illness, being psychiatrically stable, and having elevated risk for hospitalization or death. Serious mental illness is defined as having a diagnosis of schizophrenia, schizoaffective disorder, bipolar disorder, recurrent major depression with psychosis, or chronic severe post-traumatic stress disorder. Psychiatric stability is defined as having a Milestones of Recovery Scale (MORS) [20] score of 6 or greater, as assessed by the patient’s mental health clinician. Elevated risk for hospitalization is defined as having a Care Assessment Need (CAN) score greater than 75th percentile [21, 22] or medical (non-psychiatric) emergency department visit or inpatient stay within the past 6 months. CAN estimates the percentile risk of hospitalization or death within 90 days, and is computed routinely for all VA patients. Exclusion criteria include a psychiatric hospitalization within the past 6 months, receiving palliative care, being street homeless, or already receiving primary care from a specialty PACT (e.g., infectious disease PACT or home-based primary care) except for Homeless PACT, Post Deployment PACT or Women’s Health PACT.

All enrolled patients complete a 60-min in-person survey at baseline and one year later. A subset of patients at the intervention site (*n* = 30) complete an additional 20-min qualitative interview. A sample of clinicians and administrators involved with the care of the patient population are also enrolled at each site. These include staff providing care at routine PACTs, SMI PACT (intervention site) or PC-MHI (control sites), and mental health clinics. Clinicians and administrators complete a 20-min qualitative interview at baseline and 1-year follow-up.

### Settings

Outpatient mental health clinics are multidisciplinary, and staffed by psychiatrists, psychologists, nurses, social workers, and psychiatric technicians. Clinics offer a range of short- and long-term treatment services including individual, group, and family therapy; psychiatric consultation and evaluation; psychological assessment; medication management; and case management. The range of psychiatric illnesses treated cover various degrees of severity, from adjustment disorders to severe psychoses and mood disorders. Primary Care clinics operate in a multidisciplinary PACT model that includes teamlets with staffing as described earlier, protocols for communication, daily huddles, and PC-MHI.

### Implementation

To guide implementation, we use the Consolidated Framework for Implementation Research (CFIR) [23]. The CFIR is composed of five major dimensions: intervention characteristics, outer setting, inner setting, characteristics of the individuals involved, and the process of implementation. Intervention characteristics include intervention source, evidence strength, relative advantage, adaptability, trialability, complexity, design packaging, and cost. The outer setting involves patient needs and resources, cosmopolitanism, peer pressure, and external policy and incentives. The inner setting consists of structural characteristics, networks and communications, organizational culture, implementation climate, and readiness for implementation. Characteristics of individuals include knowledge and beliefs about the intervention, self-efficacy, individual stage of change, individual identification with the organization, and other personal attributes. Because SMI-PACT is an evolving approach to tailoring PACT for a specialty population, we focus on intervention characteristics, characteristics of individuals, and implementation process while examining the other characteristics more modestly. Implementation process involves 4 stages: planning, engaging, executing, and reflecting/evaluating. While here we allude below to several stages being ‘complete’, for the duration of the study we revisit each stage and re-evaluate throughout the course of implementation [23].

#### Planning

In the planning stage, the behavior and tasks for implementing SMI-PACT are developed and the quality of the model assessed [23]. During this period, an Implementation Team [24] is formed at the intervention site and conducts monthly in-person meetings. This Implementation Team includes primary care administrators, mental health administrators, systems redesign experts; and the study Principal Investigator (PI), co-PI, and Evaluation Lead. Strategic planning shapes implementation based on local structures, priorities, and patient needs. During the planning stage, the team meets regularly to tailor the model to the intervention site. Site visits allow the team to analyze facilitators and barriers to SMI-PACT implementation. Staff are enrolled across all sites.

#### Engaging

In the engaging stage, the project gathers appropriate individuals for implementation and use of the intervention [23]. In addition to teamlet members, PACT experts and clinicians are invited to several Implementation Team meetings to serve as SMI-PACT opinion leaders. These meetings continue and focus on the adaptation and implementation of the model. The team names the final teamlet composition for the intervention site. The nurse care manager is trained in treatment guidelines for prevention and medical care targets, mental illness and substance use disorders, and management of smoking and obesity. For the duration of the intervention, members of the SMI-PACT teamlet continue to receive additional training to address the needs of their patient population (e.g., motivational interviewing). Protocols operationalizing patient referrals between PACT and SMI-PACT are finalized. Communication routines within the SMI-PACT teamlet and between its partners are established, practiced, and adjusted. Throughout this period, the Evaluation Lead takes ethnographic field notes. For the remainder of the project, the team continues to foster a supportive organizational culture for SMI-PACT and addresses constraints to its implementation. The team develops patient handouts with “tips” including names and contact information for teamlet members, encouragement to use phone, or secure messaging, and availability of same-day appointments. Team members are educated regarding the importance of this activity, their role in the project, and tools they can use to motivate clinicians and facilitate implementation.

#### Executing

In the executing stage, the intervention is implemented [23]. The Implementation Team focuses on barriers and facilitators to implementation, with an eye to sustainability. The nurse care manager oversees day-to-day SMI-PACT teamlet implementation to ensure that care is coordinated, continuous, and appropriate. The nurse care manager and primary care clinician report any barriers to implementation that occur between team meetings. The PI and the co-PI assist in problem-solving and effective utilization of quality improvement techniques. Ultimately, the team guides implementation of the model and is responsible for addressing barriers. Throughout implementation, periodic and targeted feedback to the team continues and tailoring of the model proceeds accordingly.

#### Reflecting and evaluating

In the reflecting and evaluating stage, quantitative and qualitative feedback about implementation is provided [23]. Reflecting and evaluating runs concurrent with the other 3 phases, with regular consult from the team, whose input will reflect patient and clinician feedback.

### Usual care condition

The comparison condition consists of treatment as usual. The control sites continue with providing primary care and psychiatric care through PACT and specialty mental health clinics.

### Measures

#### Patient quantitative survey (baseline and 1-year follow-up)

Surveys at baseline and one year provide data on intervention impact and cost. At all sites, Research Assistants conduct baseline surveys with patients while the study co-PI provide training and oversight. Demographic data are collected at baseline, including age, race, ethnicity, and psychiatric illness history. The Service Use and Resources Form (SURF) is administered at baseline and 1-year to collect information on inpatient and outpatient service utilization for psychiatric and medical issues for visits both inside and outside VA settings. SURF has been successfully used to assess costs and service utilization with patients with SMI [25].

Three measures administered at baseline and 1-year assess patients’ experiences with SMI-PACT or usual care. The Ambulatory Care Experiences Survey (ACES) details patients’ experiences across five domains: care access, quality of doctor-patient interactions, shared decision-making, coordination of care, and helpfulness of physician office staff [26]. The Patient Assessment of Chronic Illness Care (PACIC) assesses the extent to which patients with chronic medical illness receive care that aligns with the Chronic Care Model, i.e., care that is patient-centered, proactive, planned and includes collaborative goal setting, problem-solving, and follow-up support [27]. The Client Satisfaction Questionnaire (CSQ-8) is used to assess patient satisfaction with services [28].

Patient characteristics are examined. The Behavior and Symptom Identification Scale Revised (BASIS-R) is used to assess patients’ mental health symptomatology [29]. The 13-item Patient Activation Measure (PAM 13) assesses patient knowledge, skill, and confidence for self-management of their health conditions [30]. Health related quality of life is assessed using the Veterans 12 Item Health Survey (VR-12) [31], and cognitive functioning assessed using the Hopkins Verbal Learning Test-Revised (HVLT-R) [32] and the Digit Symbol Substitution Test (DS) [33].

#### Qualitative interviews and implementation data

Semi-structured qualitative interviews are completed with intervention and control site staff. Interviews examine usual practice, and knowledge, attitudes, and behaviors regarding medical care of patients with SMI. Clinicians are asked about their expectations for SMI-PACT, and barriers and facilitators to its implementation. Administrators are interviewed to capture their perceptions of intervention characteristics, outer setting, inner setting, and implementation process. Semi-structured interviews are conducted with a subsample of patients at 1-year follow-up to assess perceptions of, acceptability of, and satisfaction with SMI-PACT.

Ethnographic field notes are also taken throughout implementation to capture aspects of the inner setting and otherwise unmeasured aspects of usual care. The SMI-PACT care manager is trained to record observational logs, and records noteworthy occurrences and events related to barriers and facilitators to implementation. Substantive emails and other communications regarding SMI-PACT implementation are archived and analyzed. Degree of implementation of SMI-PACT model components are rated as to the degree of implementation (structural changes, point-of-care changes, patient outreach changes, and management changes). Ratings are made on a 4-point scale (not at all implemented, partially implemented, mostly implemented, fully implemented).

#### Healthcare costs

Using micro-costing methods appropriate for the VA context [34] we document the cost of the SMI-PACT intervention in addition to the costs of all other health care utilized by SMI-PACT patients during the study, and similarly document costs for patients assigned to usual care. For each patient, we include costs for inpatient, outpatient, emergency department, pharmaceutical, and labs utilization during the enrollment period. Costs of non-VA services are derived from an appropriate pricing schedule, such as the Medicare fee schedule. For prescription medication costs and lab tests, we use cost estimates based on VA acquisition costs and workload.

#### Administrative data

Composite scores for diabetes mellitus control, cardiovascular health, and preventive medical services are calculated from patient record data and VA databases after 1-year follow up. These scores comprise multiple dichotomous variables concerning the quality of preventative and chronic care services (e.g., pneumococcal immunization at age 65 or older, blood pressure at guideline goal). The composite scores used in the present study have been demonstrated to be reliable, valid, and useful as part of comprehensive assessments [35]. Medication Possession Ratios (MPRs) are calculated from 12 months of pharmacy data to assess patient’s medication adherence. MPRs assess the extent to which dispensed medications provide coverage for a given interval and have been shown to be a valid measure of adherence in people with SMI [36].

### Data management and analysis plan

#### Quantitative analyses

The data analytic strategy for the site-level controlled trial is a generalized linear mixed model (GLMM). Service utilization and outcomes measured by quality of life, patient satisfaction, and composite scales are modeled as a within subject design. SMI PACT and usual care costs are compared using both unadjusted (mean and median) comparisons and GLMM. In secondary analyses, we study if patient characteristics are associated with PACT efficacy. The total direct cost of SMI-PACT is estimated by multiplying the quantities of resources used during the intervention by unit costs, and then summing over all resource categories. Enumerated resources include labor costs as well as all intervention-related diagnostic tests, office and other medical supplies, equipment depreciation, and all overhead expenses. We estimate the costs of these resources using standard methods for approximating opportunity costs [37].

#### Qualitative analyses

Qualitative analyses are conducted using a constant comparison analytic approach [38] facilitated by ATLAS.ti, a software program that allows for fluid interaction of data across types and sources [39]. Factors facilitating and impeding SMI-PACT implementation, and strengths and weaknesses of the model as implemented are assessed. The extent to which components of the model are implemented is explored, in addition to which components are efficient and easy to incorporate into routine care. We also investigate which characteristics of care are most and least salient for patients, and assess the degree of convergence between patients’ and clinicians’ perspectives. Statistical associations between care model components and important process and outcome variables are be examined, such as treatment appropriateness and improvement in patient outcomes.

#### Sample size calculation and power analyses

Researchers previously used VA national administrative data on patients with SMI who are prescribed antipsychotic medication, and calculated the proportion who received cardiometabolic clinical assessments [13]. Based on these data, we assume baseline utilization of services of 20%. With this estimation, we can detect an increase to 29% with 80% power. The analyses for the current study require a sample size of *n* = 313 at baseline and *n* = 260 at 1 year. Regarding continuous outcome variables, this study is sufficiently powered to detect changes of approximately .35 standard deviations. To be conservative, the current study aims to enroll *n* = 340 at baseline, more than the *n* = 313 on which power is based.

### Trial status

Data collection is underway. Data analysis has not yet started.

## Discussion

This is one of the first controlled trials of the implementation and effectiveness of a patient centered primary care medical home for people with serious mental illness. It will provide information regarding the value of this strategy, and processes and tools for use in implementing this model in community healthcare settings. Results may also be applicable to other high-risk and vulnerable populations, such as the homeless or cognitively impaired elderly. The intervention is tested within real-world effectiveness conditions. This study’s hybrid implementation-effectiveness design could reveal processes and complexities associated with creating, implementing and maintaining primary care homes for vulnerable populations. Innovative features of this study include defining a strategy for recruiting patients with serious mental illness who are able to have mental health care integrated within primary care, and protocols for nurse care management and collaborative care of patients with SMI.

## Abbreviations

ACES: Ambulatory Care Experiences Survey
BASIS-R: Behavior and Symptom Identification Scale-Revised
CFIR: Consolidated Framework for Implementation
CSQ-8: Client Satisfaction Questionnaire
DS: Digit Symbol Substitution Test
GLMM: Generalized Linear Mixed Model
HVLT-R: Hopkins Verbal Learning Test-Revised
MORS: Milestones of Recovery Scale
PACIC: Patient Assessment of Chronic Illness Care
PACT: Patient Aligned Care Team
PC: Primary Care
PCMH: Patient Centered Medical Home
PC-MHI: Primary Care-Mental Health Integration
PI: Principal Investigator
SMI: Serious Mental Illness
SMI-PACT: Patient Aligned Care Team for Veterans with Serious Mental Illness
SURF: Service Use and Resources Form
VA: US Department of Veterans Affairs, Veterans Health Administration
VR-12: Veterans RAND 12 Item Health Survey
